# Neuromuscular Reactivation Through Guided Visualization Combining Mindfulness and Motor Imagery via the Yuzit App: Protocol for a Randomized Controlled Trial

**DOI:** 10.2196/87895

**Published:** 2026-05-11

**Authors:** Guillaume Zunzarren, Bertrand Garet, Sonja Cabarkapa, Jérôme Murgier

**Affiliations:** 1 Orthopedic Department, Ramsay Santé, Aguiléra Private Clinic, 21 rue de l'EstagnasBiarritz, 64200, France, 33 0586520722; 2 St Vincent's Hospital Melbourne, Melbourne, Victoria, Australia

**Keywords:** mindfulness, motor imagery, neuromuscular rehabilitation, cortical activation, persistent pain, digital app, neuroplasticity

## Abstract

**Background:**

Neuromuscular activation disorders are frequently observed in patients with musculoskeletal pain and may contribute to persistent symptoms and delayed functional recovery. Neurocognitive approaches combining mindfulness and motor imagery have been proposed to target central mechanisms involved in motor control and pain modulation. Digital health tools offer an opportunity to standardize and disseminate such interventions in real-world settings.

**Objective:**

The aim of this study is to describe a neuromuscular reactivation method combining mindfulness and guided motor imagery and its digital implementation via the Yuzit app and to report preliminary observational data from real-world use.

**Methods:**

This paper primarily presents a detailed description of the intervention and its digital delivery. The protocol consists of a brief mindfulness phase followed by guided motor imagery exercises targeting specific muscle groups. In addition, we report exploratory observational data derived from the first 30 consecutive users who completed the program in routine conditions. Data were retrospectively collected through the app, including visual analogue scale (VAS) and single assessment numeric evaluation (SANE) scores, as well as user satisfaction. Analyses were descriptive and exploratory.

**Results:**

Among the first 30 users (mean age 50, SD 15.4 y; female: n=19, 63%), preliminary observations showed a reduction in VAS scores (from 5.62, SD 1.97 to 4.00, SD 2.15) and an increase in SANE scores (from 51.8, SD 17.2 to 65.1, SD 18.9) after completion of the program. Overall satisfaction was high or very high in 83% (n=25) of users. These findings are descriptive and should be interpreted as exploratory only.

**Conclusions:**

This study describes a standardized digital intervention combining mindfulness and motor imagery for neuromuscular reactivation. Preliminary real-world observations suggest the feasibility of the approach and its potential to generate patient-reported outcome data. However, no conclusions regarding efficacy can be drawn. Further controlled studies are required to evaluate clinical effectiveness.

## Introduction

Neuromuscular activation disorders are frequently observed after trauma, surgery, or chronic pain. They manifest as difficulty in effectively recruiting certain muscle groups, resulting in weakness, movement desynchronization, and inappropriate co-contractions [1-3]. These disturbances can slow functional recovery, perpetuate pain, and reinforce fear of movement [4-6]. Neuromuscular disorders can affect all the articulations of the body but the knee remains the most studied joint regarding this concern, with growing evidence of the necessity to treat this issue [7,8].

A range of therapeutic solutions are available to practitioners to help their patients improve symptoms, but these methods can be invasive and expensive [9-11]. They also might have side effects (injections, medications, etc).

Following the current trend toward the digitalization of health care [12-14], we have developed a guided visualization method for neuromuscular reactivation, combining mindfulness and motor imagery (Yuzit° SAS [15]). This approach aims to restore coherence between intention, perception, and muscle activation by mobilizing the neurocognitive mechanisms of cortical control and cerebral neuroplasticity [16]. In a recent study, it was shown to have efficacy in relieving pain in patients with obesity [14]. Mindfulness at the beginning of the session prepares the attentional state, reduces pain-related hypervigilance, and improves introspection [17]. It creates a mental context conducive to plasticity [18]. Guided motor imagery then targets muscle perception rather than the joint: the patient visualizes the contraction and direction of movement without actually performing it, according to the guiding principle that “muscles move joints, not the other way around.” [19]

To ensure the reproducibility and accessibility of this method, it has been adapted for digital use via the Yuzit app. This platform standardizes audiovisual guidance, collects self-assessments (visual analogue scale [VAS], single assessment numeric evaluation [SANE]), and guarantees data confidentiality and security. This digital adaptation allows for structured delivery, independent practice, and seamless integration into conventional rehabilitation programs.

The objective of this study was to provide a comprehensive description of a neuromuscular reactivation method combining mindfulness and guided motor imagery, along with its digital implementation via the Yuzit app. In addition, exploratory observational data from early users were analyzed to assess feasibility and the type of patient-reported outcomes that can be collected in real-world conditions. We hypothesized that use of the Yuzit app would be associated with a reduction in pain intensity and high levels of patient satisfaction.

## Methods

### Study Design

This study primarily aims to describe a novel neuromuscular reactivation method combining mindfulness and motor imagery, as well as its digital implementation via the Yuzit app. In addition to this methodological description, we report exploratory observational data derived from the first 30 consecutive users who completed the program in real-world conditions. These data were collected retrospectively during routine use of the app and were not part of a prespecified prospective protocol. Accordingly, these findings are presented for descriptive and hypothesis-generating purposes only. Users of the app voluntarily provided anonymized data during routine use, including demographic characteristics, clinical history, and self-reported outcomes (VAS and SANE scores). Data were collected at baseline, during the program, and at completion. The exploratory dataset includes the first 30 consecutive users who completed the program. The following inclusion and exclusion criteria were used (Table 1).

**Table 1.** Inclusion and exclusion criteria.

Inclusion criteria	Exclusion criteria
Adults using the Yuzit app	Incomplete data entries
Completion of the full program	Noncompletion of the program
Availability of baseline and follow-up visual analogue scale/single assessment numeric evaluation scores	Missing outcome data
Consent for use of anonymized data	Refusal to use data

### Outcomes

#### Overview

The app enables the collection of patient-reported outcomes including pain intensity (VAS), functional perception (SANE), and user satisfaction. These outcomes are reported descriptively in this manuscript and are intended to inform the design of future controlled studies. This method description study describes a new health app that was released in September 2025 (Yuzit° SAS), including the very first clinical results from the first 30 patients who completed the treatment. The analysis helps to provide initial feasibility and usability insights, illustrating the types of outcomes that can be collected via the platform and supporting the design of future controlled studies. Each session aims to reactivate conscious muscle control without physical movement. It is structured in two successive phases, delivered by an instructional voice recording to maintain concentration and engagement. The patient first selects the joint area they wish to treat via the Yuzit app on their smartphone or tablet. A program of 5 to 6 sessions is then generated. This therapy unfolds in several phases.

#### Phase 1: Mindfulness (2-3 Minutes)

The patient, comfortably positioned (seated or lying down), closes their eyes and follows verbal guidance focused on muscle groups, tone, and weight-bearing body parts. The goal is to improve concentration, reduce attentional noise, and create a state conducive to plasticity and motor learning. The patient needs to be in a quiet environment to avoid any noise disturbance.

#### Phase 2: Guided Motor Imagery

A short video presents a specific movement (eg, straight leg raise, fist clenching, hip abduction). The patient observes the movement, focusing their attention on the motor groups, and then visualizes the movement with their eyes closed, without actually performing it. Instructions focus on the sensation of contraction, the direction of force, and the continuity of the movement. Each session includes several cycles of visualization (3 to 6 movements). The actual movement is never performed during the session to avoid any compensation and preserve the purity of the cortical command.

#### Practical Settings

The intervention consisted of a digital program delivered through a smartphone app. The program included 6 sessions for programs targeting the knee, shoulder, and hip, and 5 sessions for programs targeting the ankle, cervical spine, elbow, and hand. Participants were advised to complete 2 sessions per week, although they were allowed to perform additional sessions depending on their availability. Each session included guided motor imagery exercises combined with attentional focus on bodily sensations, aiming to promote neuromuscular activation of the selected body area. Session duration ranged from 19 to 49 minutes depending on the movements included in each session. For example, in the program targeting the knee, session durations were 36, 26, 27, 24, 25, and 29 minutes, respectively. The first session was intentionally longer to introduce several exercises and to promote a noticeable benefit from the beginning of the program, with the aim of improving patient engagement and adherence. The duration of subsequent sessions depended on the movements included. Some movements required longer visualization periods than others, which explains the differences in session duration. The overall structure of the sessions was standardized, while the movements performed were adapted to the body area selected within the app. Moreover, VAS and SANE scores were recorded directly within the app at day 0, prior to session 3, and after session 5, allowing the practitioner to monitor patient progress.

#### Digital Transposition – Yuzit App

The Yuzit app was designed to standardize and secure the method while promoting patient autonomy. The simple design makes it easy to use and ensures minimal visual distractions to help optimize the patient’s ability to concentrate on auditory instructions. Moreover, it allows patient to perform this treatment at home whenever it suits them. Patients had to select the affected anatomical area (shoulder, knee, hip, spine, etc), the affected side, and the type of disorder (posttraumatic, postsurgical, persistent pain). Then, they had access to the corresponding audiovisual guidance.

#### Data Collected

Users provided the information shown in Table 2.

**Table 2.** Collected data.

Variable category	Data collected
Demographics	Age, sex, height, weight
Clinical history	Duration of symptoms (<3 months, 3-12 months, >1 year)
Pain origin	Injury, work-related, sport, unknown
Prior treatments	Physiotherapy, injections, surgery, etc
Medication use	Never, occasional, regular, daily

#### Protection and Compliance

Data were anonymized and hosted on OVH France servers certified under the French Health Data Hosting standard. The audiovisual content has been legally protected to guarantee its authenticity. Yuzit is a Class I software medical device that complies with the requirements of Regulation (EU) 2017/745 on medical devices and is Conformité Européenne–marked. It complements conventional care and allows for continued rehabilitation at home in a secure, standardized, and traceable format.

The Yuzit app is available on both iOS and Android platforms. Patients have to pay to get access to all content.

### Ethical Considerations

This study was conducted as a pragmatic evaluation of a digital health app used in routine clinical practice. According to applicable institutional and national regulations [20], formal approval from an ethics committee or institutional review board was not required because the study involved the analysis of routinely collected, noninterventional data and did not modify patient management. Therefore, the study falls within the category of research using anonymized data collected in the context of usual care. All participants were informed about the use of the app and the collection of their data for evaluation purposes prior to participation. By using the app and completing the questionnaires, participants provided informed consent for the use of their anonymized data for research and publication. All data collected through the app were anonymized prior to analysis. No personally identifiable information was included in the dataset used for the study, and appropriate safeguards were implemented to ensure the confidentiality and security of participant information. Participants did not receive any financial compensation for their participation in the study.

### Statistics

Continuous variables were described using means and standard deviations. Normality of pre–post changes was assessed using the Shapiro-Wilk test. Differences between baseline and postintervention outcomes were analyzed using paired 1-tailed *t* tests. A significance level of *P*<.05 was considered statistically significant. The minimal clinically important difference was estimated using a distribution-based approach, defined as 0.5 times the standard deviation of the change scores. The standard deviation of the change was calculated assuming a pre–post correlation coefficient of 0.7.

## Results

We assessed the first 30 patients who used the app for musculoskeletal pain. They all completed the treatment. The mean age was 50 (SD 15.4) years, the average BMI was 24.8 (SD 4.5) kg/m², and more females were included in the study than males (n=19, 63% female). The patients started the program between October 3, 2025, and October 26, 2025. The final assessments were carried out between October 13, 2025, and December 14, 2025. Patients completed the treatment after 26.1 (SD 21) days on average. The results showed an average decrease of 1.62 points in the VAS score from 5.62 (SD 1.97) to 4.00 (SD 2.15) (ie, a 29% decrease; *P*<.05) and an increase of 13.3 points in the SANE score from 51.8 (SD 17.2) to 65.1 (SD 18.9) (ie, a 26% increase; *P*=.02). These patients complained of pain in different body areas (hip, knee, ankle, shoulder, elbow, hand/wrist, lower back, and neck). The overall satisfaction was high and very high for 83% (n=25) of the patients. Their mean satisfaction score was 4.1/5.

## Discussion

Preliminary observations from the first 30 users of the app suggested a reduction in pain intensity and an improvement in perceived function after completion of the program. Pain scores decreased by an average of 1.62 points on the VAS, while SANE scores increased by 13.3 points. In addition, overall user satisfaction was high, with 83% (n=25) of participants reporting high or very high satisfaction with the intervention. These findings should be interpreted cautiously, as they were derived from an observational cohort without a control group, but they nevertheless suggest that this neurocognitive approach may represent a promising complementary strategy in musculoskeletal rehabilitation. The originality of the proposed protocol lies primarily in the explicit focus on muscle perception during motor imagery and in its standardized digital delivery. The authors have previously published a score to assess muscle perception (the Biarritz Activation Score-Knee [BAS-K]) and use it routinely [21]. While mindfulness and motor imagery have both been studied separately in rehabilitation contexts, their sequential integration within a structured and reproducible digital program represents a novel attempt to facilitate neuromuscular reactivation through cortical mechanisms. Motor imagery has been widely investigated as a strategy to activate cortical motor networks without physical movement [22]. Neuroimaging studies have shown that imagined movements activate neural circuits similar to those involved in actual movement execution, including the premotor cortex, supplementary motor area, and primary motor cortex [23]. Such activation is believed to contribute to neuroplastic changes that may facilitate motor learning and recovery. Previous works have therefore proposed motor imagery as a useful adjunct to rehabilitation, particularly in situations where physical movement is limited or painful [24,25]. In the context of musculoskeletal disorders, impaired neuromuscular activation is frequently observed following trauma, surgery, or prolonged pain states [5]. Arthrogenic muscle inhibition, for example, has been extensively described in knee injuries and can persist despite adequate structural healing [26]. These central inhibitory mechanisms may prevent effective muscle recruitment and delay functional recovery [27]. Interventions targeting cortical control and sensorimotor integration may complement conventional mechanical or strength-based rehabilitation strategies [28].

The protocol incorporates a mindfulness phase prior to imagery practice. Mindfulness interventions have been shown to modulate attention, reduce pain-related hypervigilance, and improve interoceptive awareness [29,30]. These mechanisms may create a cognitive state more favorable to motor imagery practice by enhancing concentration and reducing competing sensory inputs. Digital health platforms have also increasingly been explored as tools for delivering rehabilitation interventions [8]. Mobile apps may improve accessibility, support self-management, and enable structured home-based training. However, many currently available digital rehabilitation tools focus primarily on exercise guidance or symptom monitoring [21,31]. The Yuzit app differs in that it delivers a standardized neurocognitive intervention specifically targeting neuromuscular activation through guided imagery and attentional training.

### Strengths

One of the strengths of this study is the detailed description of a reproducible protocol combining mindfulness and motor imagery within a digital platform. The use of standardized audiovisual guidance ensures consistent delivery of the intervention across users, which may improve reproducibility compared to purely therapist-guided imagery techniques. Another strength lies in the pragmatic design of the study. The app was evaluated under real-world conditions, allowing users to continue their usual treatments while completing the program. This approach reflects typical clinical practice and may therefore provide an initial indication of the feasibility and acceptability of the intervention in routine care settings. Furthermore, the integration of outcome measures directly within the app enables automated collection of patient-reported outcomes such as VAS and SANE scores. This digital monitoring may facilitate longitudinal tracking of patient progress and support future large-scale data collection.

### Limitations

Several limitations should be acknowledged when interpreting the findings of this study. First, the sample size was relatively small and included only the first 30 users who completed the program. The absence of a control group prevents any causal inference regarding the effectiveness of the intervention. Second, participants presented with musculoskeletal pain affecting different anatomical regions, including the knee, hip, shoulder, spine, and upper limb. Although this heterogeneity reflects real-world use of the app, it also limits the ability to draw conclusions for specific clinical populations. Third, the study relied exclusively on self-reported outcome measures collected through the app. Objective assessments of neuromuscular activation, functional performance, or biomechanical changes were not included. Future studies should incorporate objective measurements to better understand the mechanisms underlying potential improvements. Another limitation concerns adherence verification. Because the intervention is performed at home, the absence of physical movement during imagery sessions cannot be directly monitored. Compliance with the instructions therefore relies on participant self-report. In addition, there was no control group due to the fact that it was a protocol study. Finally, the potential influence of concurrent treatments cannot be excluded. Participants were allowed to continue their usual therapies, which may have contributed to the observed improvements.

### Future Directions

Further research is required to evaluate the efficacy of this approach more rigorously. Randomized controlled trials comparing the Yuzit protocol with standard rehabilitation or with isolated motor imagery interventions would help determine its specific contribution to pain reduction and functional recovery. Future studies could also explore objective outcomes such as electromyographic activity, muscle activation timing, or movement biomechanics in order to better characterize the neurophysiological mechanisms involved. In addition, larger cohorts would allow subgroup analyses according to anatomical region, symptom duration, or patient characteristics. Such analyses could help identify patient populations most likely to benefit from this type of intervention. From a digital health perspective, the app also offers opportunities for further development. Integration of adherence monitoring, symptom tracking, and personalized program adjustments may enhance the effectiveness and usability of the platform. In the longer term, the use of aggregated anonymized data could also support large-scale observational research on neuromuscular rehabilitation strategies.

### Planned Statistical Analysis (Future Studies)

#### Analysis Population and Outcomes

Future prospective studies will include all eligible participants meeting predefined inclusion criteria. The primary analysis will follow an intention-to-treat approach, including all enrolled participants. A per-protocol analysis will be conducted as a sensitivity analysis. Primary outcomes will include changes in VAS and SANE scores. Secondary outcomes will include adherence, satisfaction, and usage metrics derived from the app.

#### Statistical Methods

Continuous variables will be summarized using means and standard deviations or medians and interquartile ranges, depending on distribution. Between-group comparisons in future controlled studies will be performed using appropriate parametric or nonparametric tests. All statistical tests will be 2-tailed, with a predefined significance level (eg, *α*=.05). Future studies will include formal sample size calculations based on expected clinically meaningful differences in primary outcomes (eg, VAS score changes). Where applicable, sensitivity analyses will be conducted to assess the impact of adherence, missing data, and protocol deviations on study outcomes. Missing data will be assessed for pattern and mechanism. If data are missing at random, multiple imputation techniques will be considered. Sensitivity analyses will be conducted to evaluate the robustness of findings.

#### Subgroup Analyses

Exploratory subgroup analyses will be conducted based on anatomical region, duration of symptoms, and baseline severity. These analyses will be considered hypothesis-generating.

### Conclusions

This study describes a standardized digital intervention combining mindfulness and motor imagery for neuromuscular reactivation. Preliminary real-world observations suggest the feasibility of the approach and its potential to generate patient-reported outcome data. However, no conclusions regarding efficacy can be drawn. Further controlled studies are required to evaluate clinical effectiveness.

## Abbreviations

BAS-K: Biarritz Activation Score-Knee
SANE: single assessment numeric evaluation
VAS: visual analogue scale
